# Venlafaxine in oncology: supportive-care evidence and emerging preclinical anticancer signals: a systematic review

**DOI:** 10.3389/fphar.2026.1945568

**Published:** 2026-10-16

**Authors:** Ahmad J. Harahsheh, Zhuldyz Myrzabay, Nazym Askambayeva, Reezal Ishak, Mohamad Aljofan

**Affiliations:** 1 Department of Biology and Biotechnology, Faculty of Science, The Hashemite University, Zarqa, Jordan; 2 Department of Biomedical Sciences, School of Medicine, Nazarbayev University, Astana, Kazakhstan; 3 Institute of Medical Science Technology (UniKL MESTECH), Universiti Kuala Lumpur, Kajang, Selangor, Malaysia; 4 National Laboratory Astana, Center for Life Sciences, Nazarbayev University, Astana, Kazakhstan

**Keywords:** anticancer activity, chemotherapy-induced peripheral neuropathy, drug repurposing, hot flashes, supportive oncology, venlafaxine

## Abstract

**Background:**

Venlafaxine, a serotonin–norepinephrine reuptake inhibitor, has been investigated in oncology for several supportive care indications and, more recently, for potential anticancer activity. However, the available evidence remains fragmented across clinical and preclinical studies.

**Objective:**

This systematic review aimed to synthesize the available evidence regarding the role of venlafaxine in oncology, including established supportive care applications and emerging anticancer potential.

**Methods:**

A systematic literature search was conducted in PubMed, Scopus, and Web of Science from database inception to 22 August 2026. Original clinical and preclinical studies evaluating venlafaxine in cancer populations or experimental cancer models were eligible. Clinical and preclinical evidence was synthesized separately using a narrative approach. Methodological quality was assessed using Joanna Briggs Institute tools for clinical studies and the SYRCLE Risk of Bias tool for animal studies.

**Results:**

25 studies were included, comprising 23 clinical studies and 2 preclinical studies. The strongest clinical evidence supported venlafaxine for treatment-related hot flashes, particularly among breast cancer survivors receiving endocrine therapy, with the strongest randomized, placebo-controlled trial evidence showing significantly greater reductions with venlafaxine than placebo (P < 0.0001 for higher doses); within-group percentage reductions are also reported but should not be interpreted as treatment effects. Evidence for chemotherapy-induced peripheral neuropathy was inconsistent, with potential benefit for acute oxaliplatin-related neurotoxicity but insufficient support for prevention or routine treatment of chronic CIPN. Evidence for other supportive care outcomes, including oral mucositis-associated pain, fatigue, sleep quality, depressive symptoms, and quality of life, was limited. Venlafaxine was generally well tolerated, although adverse effects varied by dose and comparator. Preclinical studies investigated anticancer activity, but not equivalently: one in melanoma models showed direct antiproliferative/pro-apoptotic effects of venlafaxine via JNK1/2–Nur77 signaling, whereas a colorectal cancer study showed only context-dependent antagonism of norepinephrine-driven tumor progression, with no significant effect of venlafaxine alone. No clinical evidence currently supports venlafaxine as a direct anticancer treatment.

**Conclusion:**

Venlafaxine has an established evidence-based role in supportive oncology for treatment-related hot flashes, whereas its role in CIPN and other supportive care indications remains uncertain. Emerging anticancer findings are limited to preclinical models and should be considered hypothesis-generating. Further randomized clinical trials are needed for supportive care indications, while additional mechanistic and preclinical validation is required before venlafaxine can be considered for clinical evaluation as an anticancer therapy.

## 1 Introduction

Cancer remains one of the leading causes of morbidity and mortality worldwide despite substantial advances in early detection and treatment. According to the latest cancer statistics, more than two million new cancer cases and over 600,000 cancer-related deaths are expected to occur in the United States in 2024 (Siegel et al., 2024). Improvements in surgery, radiotherapy, systemic therapy, targeted therapy, and immunotherapy have substantially increased survival across many malignancies, resulting in a rapidly expanding population of cancer survivors. Consequently, contemporary oncology has shifted beyond prolonging survival toward optimizing long-term health, quality of life, and survivorship care.

As survivorship continues to improve, supportive oncology has become an essential component of comprehensive cancer care. Although modern anticancer therapies have significantly improved clinical outcomes, they are frequently accompanied by acute and long-term toxicities that negatively affect physical functioning, psychological wellbeing, treatment adherence, and health-related quality of life. Persistent adverse effects, including vasomotor symptoms, chemotherapy-induced peripheral neuropathy (CIPN), fatigue, chronic pain, anxiety, depression, and cognitive impairment, remain common among cancer survivors and represent an important unmet clinical need requiring effective supportive care interventions (Lustberg et al., 2023; Silveira et al., 2026).

Venlafaxine is a serotonin–norepinephrine reuptake inhibitor (SNRI) widely prescribed for major depressive disorder and anxiety disorders. Beyond its psychiatric indications, venlafaxine has attracted increasing interest in oncology because of its potential to alleviate several treatment-related complications. The strongest clinical evidence supports its use for the management of treatment-related hot flashes, particularly among patients receiving endocrine therapy for breast cancer, where nonhormonal therapeutic options are preferred. In addition, venlafaxine has been investigated for the management of CIPN, one of the most common and debilitating toxicities associated with neurotoxic chemotherapy. Depending on the chemotherapeutic agent used, CIPN affects approximately 19%–85% of patients and frequently results in dose reduction, premature treatment discontinuation, persistent neurological dysfunction, and impaired quality of life. Venlafaxine has also been evaluated for oral mucositis-associated pain, depressive symptoms, and quality-of-life improvement, although the evidence supporting these applications remains inconsistent (Ma et al., 2025; Loprinzi et al., 2020; Tao et al., 2025; Al et al., 2024).

More recently, growing interest in drug repurposing has stimulated investigation of established non-oncologic medications as potential anticancer therapies. Drug repurposing offers several advantages over traditional drug development, including well-characterized pharmacokinetic and safety profiles, lower development costs, and shorter timelines for clinical translation. As a result, repositioning approved drugs has emerged as an attractive strategy for accelerating the discovery of novel cancer therapeutics (Al et al., 2024).

Despite the expanding literature, evidence regarding the role of venlafaxine in oncology remains fragmented. Most published studies have focused on individual supportive care indications, whereas emerging preclinical investigations have suggested that venlafaxine may also possess direct antitumor properties. However, no previous systematic review has comprehensively synthesized both the established clinical applications of venlafaxine in supportive oncology and the emerging evidence regarding its potential anticancer activity.

Therefore, the aim of the present systematic review was to critically evaluate the available clinical and preclinical evidence regarding the role of venlafaxine in oncology, summarize its efficacy across supportive care indications, assess the methodological quality of the available studies, and discuss its potential as a candidate for future cancer drug repurposing.

## 2 Methods

### 2.1 Study design

This systematic review was conducted in accordance with the Preferred Reporting Items for Systematic Reviews and Meta-Analyses (PRISMA 2020) statement. This review aimed to comprehensively evaluate both the established supportive care applications and the emerging anticancer potential of venlafaxine in oncology. Because evidence on the direct anticancer effects of venlafaxine remains limited, we considered both clinical and preclinical studies eligible to provide a comprehensive overview of the available evidence. An outline of the review, specifying the anticipated scope, eligibility criteria, information sources, and planned methodological approach, was circulated among the review team on 25 June 2026, prior to the start of the literature search (Supplementary File S1). However, the protocol was not prospectively registered in PROSPERO or any other international registry.

### 2.2 Literature search

A comprehensive literature search was performed in PubMed, Scopus, and Web of Science Core Collection from database inception until 22 August 2026. The search strategy combined Medical Subject Headings (MeSH), where applicable, and free-text terms related to venlafaxine and cancer/oncology, including neoplasm, tumor, malignancy, carcinoma, and named cancer types. The complete search strategies, including platform, filters, and search dates for each database, are provided in Supplementary Table S1.

Following database searching, all retrieved records were imported into Zotero reference management software, where duplicate records were identified and removed.

Two reviewers independently screened the titles and abstracts of all retrieved records. Full-text articles were subsequently assessed for eligibility according to the predefined inclusion and exclusion criteria. Any disagreements regarding study selection were resolved through discussion with the senior investigator and consensus was reached in all cases. The overall study selection process is presented in the PRISMA 2020 flow diagram (Figure 1).

**FIGURE 1:**
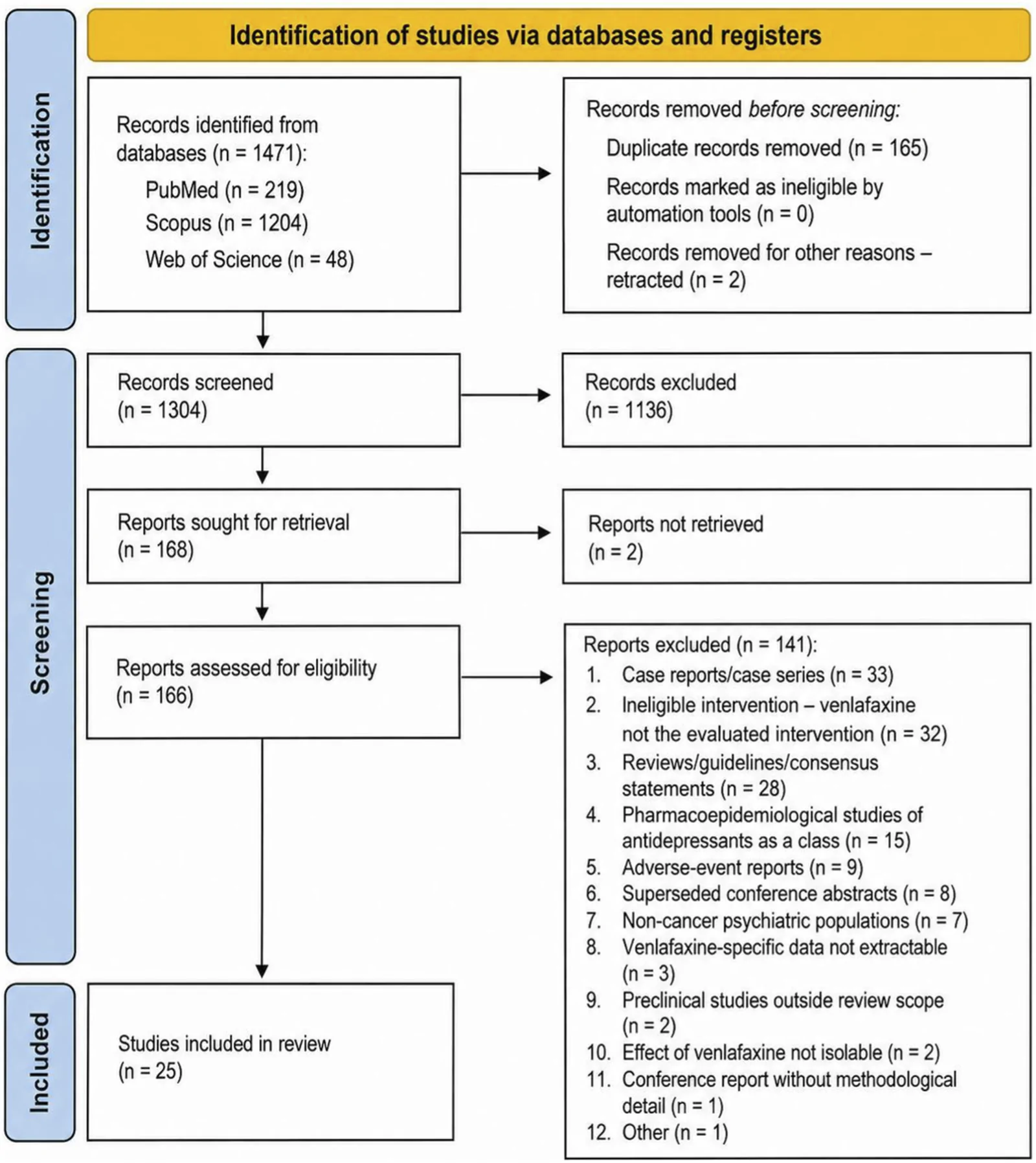
PRISMA flow diagram.

### 2.3 Eligibility criteria

Studies were considered eligible if they investigated venlafaxine in patients with malignant diseases or in experimental cancer models and evaluated either supportive care outcomes or direct anticancer effects. For vasomotor symptom (hot flash) outcomes specifically, studies enrolling mixed populations of women with a history of breast cancer and women without a cancer diagnosis who were unwilling or unable to use hormonal therapy because of breast-cancer-risk concerns were also considered eligible, reflecting the design of two influential trials from this literature that were included in this review (Loprinzi et al., 2000; Loprinzi et al., 2006). Cancer-specific results were extracted and reported separately where the original studies provided such a breakdown; where they did not, this is noted as a source of indirectness (see Section 4, Limitations). Both clinical and preclinical original research studies published in English were eligible for inclusion.

Studies were excluded if they were review articles, editorials, conference abstracts, letters, case reports, or did not report original data. Studies conducted exclusively in non-cancer populations, studies not evaluating venlafaxine, and studies in which the independent effect of venlafaxine could not be determined because of combination interventions were also excluded.

Data extraction was performed independently by the same two reviewers using a standardized data extraction form. The extracted information included the first author, year of publication, country, cancer type, study design, study population or experimental model, sample size, intervention characteristics, comparator, primary outcomes, principal findings, and proposed molecular mechanisms for preclinical studies. Any discrepancies during data extraction were resolved through discussion with the senior investigator until consensus was achieved.

### 2.4 Quality assessment

The methodological quality of the included clinical studies was independently assessed by two reviewers using the Joanna Briggs Institute (JBI) Critical Appraisal Checklists, with the checklist selected according to the individual study design. Because the review also included two preclinical animal studies evaluating the direct anticancer activity of venlafaxine, their methodological quality was assessed using the SYRCLE Risk of Bias tool, which is specifically designed for animal intervention studies. Disagreements in quality assessment were resolved through discussion with the senior investigator. The results of the quality assessment are presented in Supplementary Tables S2, S3.

### 2.5 Data synthesis

The included studies were grouped according to their primary focus: (1) management of treatment-related hot flashes, (2) chemotherapy-induced peripheral neuropathy, (3) other supportive care applications, and (4) emerging anticancer activity. Clinical findings and preclinical mechanistic evidence were synthesized separately to provide a comprehensive overview of the current evidence regarding the role of venlafaxine in cancer care.

A meta-analysis was not performed because the included studies differed substantially in comparator groups, treatment duration, dose, outcome definitions, and reporting formats. Although several studies evaluated hot flashes, they used different designs, including placebo-controlled, active-comparator, crossover, open-label, and single-arm designs, which limited the appropriateness of pooling. Therefore, results were synthesized narratively and supported by structured tabulation of study characteristics and effect estimates where available.

## 3 Results

### 3.1 Study selection

A total of 1,471 records were identified through database searching, including PubMed (n = 219), Web of Science Core Collection (n = 48), and Scopus (n = 1,204). After removing 165 duplicate records and two retracted publications, 1,304 records remained for title and abstract screening. Of these, 1,136 records were excluded during title and abstract screening, and 168 reports were sought for retrieval. Two reports could not be retrieved, leaving 166 reports for full-text eligibility assessment. Of these, 141 were excluded for predefined reasons (see Supplementary Table S4). 25 studies met the eligibility criteria and were included in the qualitative synthesis, comprising 23 clinical studies and two preclinical studies (Figure 1).

One potentially eligible recently published preclinical study (Mo et al., 2026) was identified during the updated search. However, the full-text article could not be retrieved despite attempts to obtain it. Therefore, eligibility could not be confirmed through full-text assessment, and the study was not included in the final evidence synthesis (Mo et al., 2026).

### 3.2 Characteristics of the included studies

The characteristics of the included clinical studies are summarized in Table 1. Overall, 23 clinical studies published between 1998 and 2026 were included. The majority investigated the role of venlafaxine in supportive cancer care, particularly for the management of treatment-related hot flashes, chemotherapy-induced peripheral neuropathy, and cancer-related symptom control. Most studies were randomized controlled trials, although several prospective single-arm studies and one retrospective case-control study were also identified. Sample sizes ranged from 13 to 309 participants.

**TABLE 1:** Characteristics of the included studies evaluating the role of venlafaxine in cancer care.

First author (Year)	Country	Cancer type	Study population	Study design	Sample size	Intervention	Comparator	Duration	Primary outcome(s)	Main findings
Buijs et al. (2009)	Netherlands	Breast cancer	Breast cancer patients with treatment-induced menopause experiencing hot flashes	Double-blind, randomized, crossover trial	60	Venlafaxine	Clonidine	8 weeks per treatment period with a 2-week washout	Hot flash frequency and severity, quality of life, sexual functioning, adverse events	Venlafaxine and clonidine demonstrated comparable efficacy in reducing hot flashes (49% vs. 55%, *P* = NS). Venlafaxine was associated with a higher rate of treatment discontinuation due to adverse effects
Loibl et al. (2007)	Germany	Breast cancer	Breast cancer patients experiencing ≥2 hot flashes/day	Phase III, double-blind, randomized trial	64*	Venlafaxine 37.5 mg twice daily	Clonidine 0.075 mg twice daily	4 weeks	Hot flash frequency	Venlafaxine significantly reduced hot flash frequency compared with clonidine (median reduction: 7.6 vs. 4.85 hot flashes/day; *P* = 0.025), demonstrating superior efficacy
Loprinzi et al. (2000)	United States	Breast cancer	Women with a history of breast cancer, or without breast cancer but unwilling to use hormonal therapy due to breast-cancer-risk concerns (mixed cancer/non-cancer population)	Randomized, double-blind, placebo-controlled trial	191*	Venlafaxine 37.5, 75, or 150 mg/day	Placebo	4 weeks	Hot flash frequency and hot flash score	Venlafaxine produced dose-dependent reductions in hot flash scores. The 75 mg/day and 150 mg/day doses achieved a 61% reduction compared with 27% in the placebo group, although higher doses were associated with more adverse events
Carpenter et al. (2007)	United States	Breast cancer	Breast cancer survivors experiencing hot flashes	Randomized, double-blind, placebo-controlled crossover trials	77 (57 low-dose; 20 high-dose)	Venlafaxine 37.5 mg/day or 75 mg/day	Placebo	4 weeks	Physiological and self-reported hot flashes, fatigue, sleep quality, quality of life	Venlafaxine produced modest reductions in subjective hot flashes. The 75 mg/day dose significantly reduced hot flash interference, while clinically meaningful improvements in fatigue, sleep quality, and quality of life were observed among women achieving >50% reduction in physiological hot flashes. Long-term discontinuation was common despite generally mild adverse effects
Vitolins et al. (2013)	United States	Prostate cancer	Androgen-deprived men with prostate cancer experiencing hot flashes	Randomized, 2 × 2 factorial clinical trial	120	Venlafaxine	Placebo (with or without soy protein supplementation)	12 weeks	Hot flash symptom severity score (HFSSS), quality of life	Venlafaxine did not significantly reduce hot flash symptom severity compared with placebo. Soy protein improved some quality-of-life measures, whereas venlafaxine showed no significant benefit for hot flashes in androgen-deprived men
Zimmerman et al. (2016)	United States	Colorectal cancer	Patients with stage II–III or stage IV colon cancer receiving oxaliplatin-based chemotherapy (FOLFOX)	Randomized, double-blind, placebo-controlled pilot trial	50	Venlafaxine XR 37.5 mg twice daily	Placebo	Throughout oxaliplatin treatment until the last cycle	Prevention of chemotherapy-induced peripheral neuropathy (CIPN20 sensory subscale, physician- and patient-reported neurotoxicity)	Venlafaxine did not significantly prevent oxaliplatin-induced peripheral neuropathy compared with placebo. Although modest improvements were observed in some acute neuropathy symptoms, the primary endpoint (CIPN20 sensory subscale) was not met, and the findings did not support progression to a phase III trial
Bordeleau et al. (2010)	Canada	Breast cancer	Postmenopausal breast cancer survivors with ≥14 bothersome hot flashes/week	Multicenter, randomized, open-label, crossover trial	66	Venlafaxine (37.5 mg/day for 7 days, then 75 mg/day for 21 days)	Gabapentin (300–900 mg/day)	Two 4-week treatment periods separated by a 2-week washout	Patient preference, hot flash score, toxicity	Both venlafaxine and gabapentin reduced hot flash scores by 66%. However, 68% of patients preferred venlafaxine versus 32% who preferred gabapentin (*P* = 0.01). Venlafaxine was associated with more nausea, appetite loss, and constipation, whereas gabapentin caused more dizziness and increased appetite
Boekhout et al. (2011)	Netherlands	Breast cancer	Patients with a history of breast cancer experiencing treatment-induced hot flashes	Randomized, double-blind, placebo-controlled trial	102	Venlafaxine 75 mg/day	Clonidine 0.1 mg/day; placebo	12 weeks	Daily hot flash score, sleep quality, anxiety, depression, sexual function	Over the 12-week treatment period, both venlafaxine and clonidine significantly reduced hot flash scores compared with placebo. Venlafaxine produced a more rapid initial reduction in symptoms, whereas clonidine demonstrated greater benefit by week 12. Venlafaxine was associated with higher rates of nausea, constipation, and appetite loss
Biglia et al. (2005)	Italy	Breast cancer	Breast cancer survivors with troublesome hot flashes, including patients receiving adjuvant tamoxifen	Prospective, open-label, single-arm study	40	Venlafaxine XR 37.5 mg/day	None	8 weeks	Hot flash frequency, hot flash score, depressive symptoms (BDI), tolerability	Venlafaxine significantly reduced hot flash frequency by 39% after 4 weeks and 53% after 8 weeks, while hot flash score decreased by 59% (*P* < 0.001). Treatment was well tolerated, with mostly mild nausea and dry mouth. Improvement was maintained in patients receiving tamoxifen, and depressive symptoms decreased by 23% among study completers
Irani et al. (2010)	France	Prostate cancer	Men with prostate cancer receiving androgen deprivation therapy (leuprorelin) and experiencing bothersome hot flashes	Multicenter, randomized, double-blind trial	309*	Venlafaxine 75 mg/day	Cyproterone acetate 100 mg/day; medroxyprogesterone acetate 20 mg/day	10 weeks	Daily hot flash score, quality of life, safety	Venlafaxine significantly reduced hot flash scores from baseline (−47.2%, *P* < 0.0001), but both cyproterone acetate (−94.5%) and medroxyprogesterone acetate (−83.7%) were significantly more effective than venlafaxine (*P* < 0.0001). Serious treatment-related adverse events were uncommon in all groups
Durand et al. (2012)	France	Predominantly colorectal cancer	Patients with oxaliplatin-induced acute neurotoxicity receiving oxaliplatin-based chemotherapy	Randomized, double-blind, placebo-controlled phase III trial	48	Venlafaxine (50 mg before oxaliplatin infusion, followed by venlafaxine XR 37.5 mg twice daily on days 2–11)	Placebo	11 days per chemotherapy cycle	Complete relief of acute oxaliplatin-induced neurotoxicity, neuropathic pain, oxaliplatin-specific neurotoxicity	Complete symptom relief was achieved in 31.3% of patients receiving venlafaxine versus 5.3% receiving placebo (*P* = 0.03). Venlafaxine significantly reduced acute oxaliplatin-induced neurotoxicity and was generally well tolerated, with only grade 1–2 nausea and asthenia reported
Ma et al. (2023)	United States	Head and neck squamous cell carcinoma	Patients with nonmetastatic head and neck cancer undergoing definitive chemoradiation	Single-center, phase II randomized clinical trial	62	Gabapentin (3,600 mg/day) + venlafaxine (150 mg/day)	Gabapentin (3,600 mg/day) alone	During chemoradiation	Oral mucositis pain, quality of life, opioid use, feeding tube placement, adverse events	The addition of venlafaxine to prophylactic gabapentin did not improve oral mucositis pain, quality of life, opioid use, feeding tube placement, or toxicity compared with gabapentin alone
Farshchian et al. (2018)	Iran	Mixed cancer population	Cancer patients with chemotherapy-induced peripheral neuropathy	Randomized clinical trial	156	Venlafaxine	Duloxetine; placebo	4 weeks	Cranial, sensory and motor neuropathy, neuropathic pain	Both venlafaxine and duloxetine significantly improved chemotherapy-induced peripheral neuropathy compared with baseline. However, duloxetine demonstrated greater efficacy than venlafaxine, particularly in reducing motor neuropathy and neuropathic pain (*P* < 0.05)
Walker et al. (2010)	United States	Breast cancer	Patients with hormone receptor-positive breast cancer receiving antiestrogen therapy and experiencing vasomotor symptoms	Randomized controlled trial	50	Venlafaxine	Acupuncture	12 weeks (1-year follow-up)	Hot flash frequency, quality of life, depressive symptoms, adverse events	Both venlafaxine and acupuncture significantly reduced hot flashes and improved quality of life, with comparable efficacy. However, acupuncture was associated with fewer adverse effects and more sustained symptom control after treatment discontinuation, whereas hot flashes increased after venlafaxine withdrawal
Sato et al. (2026)	Japan	Hormone receptor-positive breast cancer	Patients receiving tamoxifen with moderate-to-severe hot flashes	Prospective, single-arm, open-label study	20 (17 efficacy; 20 safety)	Venlafaxine 37.5 mg/day with optional escalation to 75 mg/day	None	4 weeks	Hot flash score, safety, tamoxifen pharmacokinetics, CYP2D6 genotype	Hot flash scores decreased by 49.8% at week 4 (*P* < 0.001). Venlafaxine did not alter serum concentrations of tamoxifen or its active metabolites, and all adverse events were Grade 1–2. Symptom improvement was observed regardless of CYP2D6 genotype
Radkhah et al. (2024)*	Iran	Breast cancer	Patients with acute taxane-induced neuropathy following paclitaxel chemotherapy	Randomized clinical trial	88	Venlafaxine	Duloxetine	10 weeks	Neuropathic pain, neuropathy severity, quality of life, anxiety, depression	Both venlafaxine and duloxetine significantly improved neuropathic symptoms. Venlafaxine produced a significant reduction in McGill pain scores, while no statistically significant differences were observed between the two treatment groups across overall outcome measures
Loprinzi et al. (1998)	United States	Mixed: Breast cancer survivors + prostate cancer (ADT)	Cancer patients/survivors with hot flashes	Phase II, single-arm, open-label	28	Venlafaxine 12.5 mg PO BID	None	1-week baseline +4 weeks	Hot flash score (frequency × severity)	58% had >50% reduction in hot flash score by week 4; median reduction 55%; well tolerated
Quella et al. (1999)	United States	Prostate cancer	Men on androgen deprivation therapy with hot flashes	Pilot trial, single-arm	16	Venlafaxine 12.5 mg PO BID	None	1-week baseline +4 weeks	Hot flash score reduction	63% had >50% reduction by week 4; median reduction 54%; severe episodes 2.3→0.6/day (p = 0.003)
Loprinzi et al. (2006)	United States	Breast cancer	Women with bothersome hot flashes	Phase III RCT	218 (109/arm)	Venlafaxine 37.5 mg/day ×1 week, then 75 mg/day	Depo-medroxyprogesterone acetate 400 mg IM ×1	6 weeks	Hot flash score/frequency reduction	DMPA superior to venlafaxine (79% vs. 55% reduction, p < 0.0001); DMPA less toxic
Kus et al. (2016)	Turkey	Mixed (breast/gynecologic/colon, taxane/oxaliplatin-treated)	Patients with CIPN, Grade 1–3	Retrospective case-control	206 (91 venlafaxine, 115 control)	Venlafaxine XR 75 mg/day	None	3 chemo cycles, assessed q3wk	>75% symptomatic relief rate (NRS)	53.5/58.3/45.2% relief in venlafaxine arm vs. 0/0/0% control across 3 visits
Taleghani et al. (2023)	Iran	Breast cancer	Women 35–65 years on tamoxifen with hot flashes	Double-blind, placebo-controlled RCT	41	Venlafaxine 37.5→75 mg; citalopram 10→20 mg	Placebo	4 weeks + 2 months follow-up	Total efficacy (%)	Citalopram 64.3% > venlafaxine 53.8% > placebo 14.3% (p = 0.02)
Tasmuth et al. (2002)	Finland	Breast cancer	Women with neuropathic pain in the anterior chest wall, axilla and/or medial upper arm following primary treatment for breast cancer; median age 55 years (range 37–72); median 20 months since surgery	Randomized, double-blind, placebo-controlled crossover trial	15 entered; 13 analyse	Venlafaxine 18.75 mg tablets, escalated weekly to a maximum tolerated dose of 37.5 mg/day (n = 2) or 75 mg/day (n = 11)	Placebo	10 weeks total (crossover)	Average daily pain intensity recorded in a diary	The primary outcome was not met: average daily pain intensity did not differ significantly between venlafaxine and placebo. Secondary outcomes favoured venlafaxine: Average pain relief (diary) was significantly greater (P < 0.05) and maximum pain intensity over the preceding week was significantly lower (P < 0.05) than with placebo. Pain intensity at rest, minimum pain intensity, anxiety and depression did not differ. Adverse effects did not differ significantly between treatments. 11/13 patients reported ≥50% pain relief
van Gool et al. (2005)	Netherlands	Breast cancer	Women with hot flushes (January 2001 – July 2003); mean age 49.2 years (range 35–65)	Prospective, open-label, single-centre clinical case series (no control group)	22 referred; 14 started venlafaxine	Venlafaxine 75 mg/day, increased to 150 mg/day if response was insufficient	None	Follow-up visits at 2–3 weeks, 4–6 weeks and 4–6 months; median follow-up 13 months (range 5–36) in responders	Self-monitored hot flush frequency (no predefined response criteria)	Of 14 women who started venlafaxine, 6 (43%) achieved a >50% reduction in hot flush frequency sustained at a median follow-up of 13 months, with complete resolution in 2. Two women did not tolerate the 75 mg starting dose; 4 reported a 30%–50% reduction but discontinued because of side effects; 2 had no effect at 75 or 150 mg. The authors emphasise substantial heterogeneity in referred patients and a higher-than-expected discontinuation rate

Two additional preclinical studies investigated the direct anticancer activity of venlafaxine using melanoma and colorectal cancer models. Both studies included *in vitro* and *in vivo* experiments and demonstrated inhibitory effects on tumor growth through distinct molecular mechanisms involving the JNK1/2–Nur77 pathway and the NET–PPP2R1A–Akt–VEGF signaling axis (Table 2).

**TABLE 2:** Characteristics of the included preclinical studies investigating the anticancer effects of venlafaxine.

Study	Cancer model	Study type	Intervention	Main findings	Proposed mechanism
Niu et al. (2023)	Human melanoma cell line (MV3), *in vitro*; MV3 xenograft in immunodeficient BALB/c nude mice, *in vivo* (conventional human tumor xenograft model)	Preclinical (*in vitro* and *in vivo*)	Venlafaxine (10 μM *in vitro*; 20 mg/kg i.p. *in vivo*)	Inhibited melanoma cell proliferation, induced apoptosis, and reduced xenograft tumor growth by approximately 64%	Activation of the JNK1/2–Nur77 signaling pathway, mitochondrial translocation of Nur77, increased ROS generation, and activation of the caspase-3/PARP apoptotic pathway
Zhang et al. (2023)	CT26 murine colon carcinoma implanted in immunocompetent BALB/c mice, *in vivo* (syngeneic model); HCT116 and SW480 human colorectal cancer cell lines, *in vitro* only (no human xenograft was used)	Preclinical (*in vitro* and *in vivo*)	Venlafaxine (10 μM *in vitro*; 10 mg/kg i.p. *in vivo*)	Venlafaxine alone did not significantly alter tumor growth/viability/VEGF/Akt/microvessel density vs. control; antagonized norepinephrine-enhanced proliferation, angiogenesis, and tumor growth (combination vs. norepinephrine-alone: 25.1% tumor volume, 28.7% tumor weight)	Inhibition of NET/SLC6A2, resulting in suppression of the NET–PPP2R1A–Akt–VEGF signaling pathway and tumor angiogenesis

#### 3.2.1 Methodological quality

We assessed the methodological quality of the included clinical studies using the appropriate JBI Critical Appraisal Checklist for each design (RCT, quasi-experimental, or case-control), and we assessed preclinical animal studies using the SYRCLE Risk of Bias tool (Supplementary Tables S2, S3). Of the 23 included clinical studies, five were rated High quality (Buijs et al., 2009; Loibl et al., 2007; Loprinzi et al., 2000; Boekhout et al., 2011; Irani et al., 2010), four were rated Moderate–High (Carpenter et al., 2007; Vitolins et al., 2013; Durand et al., 2012; Ma et al., 2023), 13 were rated Moderate (Zimmerman et al., 2016; Bordeleau et al., 2010; Biglia et al., 2005; Farshchian et al., 2018; Walker et al., 2010; Sato et al., 2026; Radkhah et al., 2024; Loprinzi et al., 1998; Quella et al., 1999; Loprinzi et al., 2006; Kus et al., 2016; Taleghani et al., 2023; Tasmuth et al., 2002), and one was rated Low (van Gool et al., 2005). Most Moderate-rated studies were single-arm, open-label, retrospective, or pilot-scale designs, for which the Moderate ceiling reflects the absence of a concurrent control group, blinding, or randomization inherent to these designs rather than deficiencies in study conduct; the exception is Tasmuth et al. (2002), a randomized, double-blind, placebo-controlled crossover trial whose rating reflects a very small analysed sample, an undescribed washout period, and analysis restricted to completers. The single Low rating reflects a clinical case series conducted without predefined inclusion or response criteria and reported descriptively without statistical analysis. For the preclinical evidence, SYRCLE ratings were Moderate for both included animal studies (Niu et al., 2023; Zhang et al., 2023). This appraisal reflects only the *in vivo* component and does not extend to the *in vitro* mechanistic experiments reported alongside it. These risk-of-bias findings were used to weight the strength of conclusions drawn in the synthesis below (Sections 3.3–3.8), with findings from High and Moderate–High rated randomized, blinded, placebo-controlled trials treated as the primary basis for efficacy conclusions, and findings from Moderate- and Low-rated studies treated as supportive and hypothesis-generating.

### 3.3 Venlafaxine for the management of hot flashes

Hot flashes represented the most extensively investigated indication for venlafaxine in oncology, accounting for the majority of the included clinical studies. Most studies enrolled breast cancer survivors receiving endocrine therapy or women with treatment-induced menopause, whereas two studies evaluated androgen deprivation-associated hot flashes in men with prostate cancer. Several randomized, placebo-controlled trials reported within-group reductions in hot-flash frequency or severity of approximately 50%–60% in venlafaxine-treated participants. However, these figures reflect change from baseline within the treatment arm and should not be interpreted as the treatment effect attributable to venlafaxine, since comparable within-group improvement was also observed in placebo arms in several of these trials. Between-group comparative effect estimates, where reported, are presented below. Two early pilot studies established the feasibility of low-dose venlafaxine for hot flash management. In an early single-arm pilot study without a concurrent control group, Loprinzi et al. reported a within-group reduction in hot flash score of greater than 50% in 58% of cancer survivors and men undergoing androgen-deprivation therapy after 4 weeks of low-dose venlafaxine (12.5 mg twice daily); the authors noted this uncontrolled design as the basis for pursuing a subsequent placebo-controlled trial (Loprinzi et al., 1998). Similarly, in a single-arm pilot trial without a placebo control, restricted to men receiving androgen deprivation therapy for prostate cancer, Quella et al. found a within-group reduction of greater than 50% in hot flash score in 63% of evaluable patients, with severe or very severe episodes decreasing from 2.3 to 0.6 per day (P = 0.003); the authors explicitly noted the absence of a placebo control as a limitation of this trial (Quella et al., 1999).

In a randomized, double-blind, placebo-controlled trial (primary endpoint: average daily hot-flash activity, met), Loprinzi et al. demonstrated a dose-dependent reduction in hot flash scores: within-group reductions from baseline were 27% (95% CI 11–34) with placebo, 37% (26–54) with 37.5 mg/day, 61% (50–68) with 75 mg/day, and 61% (48–75) with 150 mg/day, with each venlafaxine dose differing significantly from placebo (P < 0.001). Both the placebo and venlafaxine percentages are within-group changes; the significant between-group effect is reflected in the P values, not in the magnitude of the raw percentages themselves (Loprinzi et al., 2000). Biglia et al., a prospective single-arm study without a concurrent control group, reported within-group reductions in hot flash frequency (53%) and hot flash score (59%) after 8 weeks of low-dose venlafaxine (37.5 mg/day); in the absence of a comparator arm, this design cannot establish comparative efficacy (Biglia et al., 2005). More recently, Sato et al., also a single-arm study without a control group, observed a within-group reduction of 49.8% in hot flash scores among patients receiving tamoxifen, while demonstrating that venlafaxine did not alter serum concentrations of tamoxifen or its active metabolites; as an uncontrolled design, this finding is hypothesis-generating rather than confirmatory of comparative efficacy (Sato et al., 2026).

In a prospective open-label clinical series without a control group, van Gool et al. reported that 6 of 14 women with a history of breast cancer who started venlafaxine (75–150 mg/day) achieved a greater than 50% reduction in self-monitored hot flush frequency, sustained at a median follow-up of 13 months, with complete resolution in two patients (van Gool et al., 2005). However, 2 of 14 women did not tolerate the 75 mg starting dose and a further four discontinued because of side effects despite a partial (30%–50%) reduction in hot flush frequency, so this uncontrolled series also illustrates the tolerability-related attrition that may limit effectiveness in routine practice.

Several comparative studies evaluated venlafaxine against other nonhormonal interventions. Loibl et al., a double-blind RCT (primary endpoint: hot flash frequency at week 4, met), found venlafaxine superior to clonidine in reducing frequency (57% vs. 37% reduction from baseline; median drop of 7.6 vs. 4.85 hot flashes/day, P = 0.025). However, on the secondary hot-flash severity score, the direction reversed: clonidine produced a significantly greater reduction than venlafaxine (57% vs. 39%, P = 0.043) (Loibl et al., 2007). Buijs et al., a double-blind crossover trial, reported comparable efficacy between venlafaxine and clonidine, with no significant difference in hot flash score reduction (49% vs. 55% after 8 weeks, P = 0.55); venlafaxine was associated with significantly more treatment discontinuations due to toxicity (14/59 vs. 5/53, P = 0.038) (Buijs et al., 2009). Boekhout et al., a three-arm, double-blind, placebo-controlled RCT (primary endpoint: hot flash score at week 12), found that clonidine differed significantly from placebo at the primary endpoint (P = 0.03), while the difference for venlafaxine versus placebo was borderline non-significant (P = 0.07); the primary endpoint was therefore not formally met for venlafaxine. Over the full 12-week treatment course, however, venlafaxine did differ significantly from placebo (41% reduction, P < 0.001), as did clonidine (26% reduction, P = 0.045), with venlafaxine producing a faster initial reduction (42% during weeks 1–4, P = 0.01) (Boekhout et al., 2011). Bordeleau et al., an open-label crossover RCT (primary endpoint: patient preference, not hot flash score), found no significant difference in hot flash score reduction between venlafaxine and gabapentin (66% overall, ratio of effects 0.96, P > 0.61), but a significant majority of patients preferred venlafaxine (68% vs. 32%, P = 0.01) (Bordeleau et al., 2010). Walker et al., an RCT (primary endpoint: hot flash frequency), found both acupuncture and venlafaxine produced a similar 50% reduction in hot flash frequency post-treatment (significant group effect, P = 0.036; no interaction), but within 2 weeks of treatment discontinuation, hot flashes increased significantly in the venlafaxine group while remaining low in the acupuncture group (interaction P = 0.029), indicating more durable symptom control with acupuncture. Adverse events occurred in the venlafaxine group only (18 incidents vs. 0, P = 0.002) (Walker et al., 2010).

In a larger phase III trial (NCCTG N99C7), Loprinzi et al. found that depomedroxyprogesterone acetate produced a significantly greater reduction in hot flash scores than venlafaxine (79% vs. 55%, p < 0.0001), although venlafaxine avoided the risks associated with progestin exposure (Loprinzi et al., 2006). Similarly, in a placebo-controlled comparison among tamoxifen-treated women, Taleghani et al. found that citalopram achieved higher total efficacy than venlafaxine (64.3% vs. 53.8%), though both agents outperformed placebo (14.3%, p = 0.02) (Taleghani et al., 2023).

Evidence supporting venlafaxine for hot flash management in men receiving androgen deprivation therapy was less consistent. Irani et al. reported that venlafaxine significantly reduced hot flash scores from baseline but was less effective than hormonal therapies (Irani et al., 2010). Vitolins et al., a randomized, double-blind, placebo-controlled, 2 × 2 factorial trial (primary endpoint: hot flash symptom severity score), found no significant between-group difference: at week 2, the decrease was 28% with venlafaxine versus 2% without (P = 0.005), but by week 12 the decrease was 29% with venlafaxine versus 36% without (P = 0.723). The primary endpoint was not met, and the trial was stopped early by the Data Safety Monitoring Board before reaching its planned sample size of 176 (Vitolins et al., 2013).

Quantitative pooling of the breast-cancer/endocrine-therapy hot-flash studies was considered but judged not feasible for this subset as a whole, given heterogeneity in venlafaxine dose (12.5–150 mg/day), treatment duration (4–12 weeks), and outcome metric (frequency alone, severity alone, or a composite score) across trials. Pooling was not considered for the single-arm studies (Loprinzi et al., 1998; Biglia et al., 2005; Sato et al., 2026) given the absence of a comparator, nor for the androgen-deprivation population (Quella et al., 1999; Irani et al., 2010; Vitolins et al., 2013), which differs in population and comparator from the breast-cancer subset.

Overall, the strongest clinical evidence supporting venlafaxine in oncology was observed for the management of treatment-related hot flashes.

### 3.4 Venlafaxine for chemotherapy-induced peripheral neuropathy

Five clinical studies evaluated venlafaxine for chemotherapy-induced peripheral neuropathy, including both acute oxaliplatin-induced neurotoxicity and chronic or established CIPN. Overall, the evidence was heterogeneous. The most favorable findings were observed for acute oxaliplatin-related neurotoxicity, whereas evidence for prevention or treatment of chronic CIPN was inconsistent (Durand et al., 2012; Farshchian et al., 2018; Radkhah et al., 2024; Zimmerman et al., 2016).

Durand et al. reported that venlafaxine significantly improved acute oxaliplatin-induced neurotoxicity, with complete symptom relief achieved in 31.3% of patients compared with 5.3% in the placebo group (Durand et al., 2012). In contrast, Zimmerman et al. did not demonstrate a significant preventive effect of venlafaxine against chronic oxaliplatin-induced peripheral neuropathy, and the primary endpoint based on the EORTC QLQ-CIPN20 sensory subscale was not achieved (Zimmerman et al., 2016).

Consistent with these findings, in a retrospective case-control study, Kus et al. reported that venlafaxine XR (75 mg/day) achieved greater than 75% symptomatic relief in 53.5%, 58.3%, and 45.2% of patients across three sequential visits, compared with 0% in the untreated control group (Kus et al., 2016).

Comparative studies with duloxetine also yielded mixed findings. Farshchian et al. observed that both venlafaxine and duloxetine significantly improved chemotherapy-induced peripheral neuropathy, although duloxetine showed greater efficacy, particularly in reducing motor neuropathy and neuropathic pain (Farshchian et al., 2018). Similarly, Radkhah et al. reported significant improvements in neuropathic symptoms in both treatment groups, with no statistically significant differences observed between venlafaxine and duloxetine across overall clinical outcomes, despite greater reductions in McGill pain scores among patients receiving venlafaxine (Radkhah et al., 2024).

### 3.5 Venlafaxine for treatment-related neuropathic pain following breast cancer surgery

One randomized, double-blind, placebo-controlled crossover trial evaluated venlafaxine for neuropathic pain persisting after primary treatment for breast cancer. Tasmuth et al. randomized 15 women with neuropathic pain of the anterior chest wall, axilla, and/or medial upper arm; 13 were analysed after titration to a maximum tolerated dose of 37.5 or 75 mg/day (Tasmuth et al., 2002). The primary outcome, average daily pain intensity recorded by diary, did not differ significantly between venlafaxine and placebo. Among secondary outcomes, average pain relief was significantly greater with venlafaxine (P < 0.05) and maximum pain intensity during the preceding week was significantly lower (P < 0.05), whereas pain intensity at rest, minimum pain intensity, anxiety, and depression did not differ between treatments. Adverse effects were reported with similar frequency and severity in both treatment periods. 11 of the 13 analysed patients reported at least 50% pain relief.

### 3.6 Other supportive care applications

Only one trial, Ma et al., was specifically designed to evaluate a distinct supportive-care indication, oral mucositis-associated pain, as a primary outcome. Fatigue, sleep quality, depressive symptoms, and quality of life were not independently investigated; they were assessed only as secondary or exploratory outcomes within trials primarily designed to evaluate hot flashes, and in one case only within a responder subgroup. These should not be interpreted as independent evidence for these indications.

In a randomized phase II clinical trial, Ma et al. evaluated the addition of venlafaxine to prophylactic high-dose gabapentin for patients with head and neck squamous cell carcinoma receiving definitive chemoradiotherapy (Ma et al., 2023). The addition of venlafaxine did not significantly improve oral mucositis pain, quality of life, opioid consumption, feeding tube placement, or treatment-related toxicity compared with gabapentin alone.

Several hot flash studies additionally assessed secondary patient-reported outcomes. Carpenter et al. observed improvements in fatigue, sleep quality, and quality of life as a secondary, exploratory analysis restricted to the subgroup of women who achieved clinically meaningful reductions in physiological hot flashes (Carpenter et al., 2007), not the full trial population. Likewise, Biglia et al. found reductions in depressive symptoms following 8 weeks of low-dose venlafaxine treatment (Biglia et al., 2005). However, improvements in these secondary supportive care outcomes were not consistently reported across all studies.

### 3.7 Safety and tolerability

Across clinical studies, venlafaxine was generally well tolerated, although adverse effects varied by dose and comparator. The most frequently reported adverse events included nausea, constipation, appetite loss, dry mouth, dizziness, asthenia, and treatment discontinuation due to intolerance in some trials (Loprinzi et al., 2000; Biglia et al., 2005; Boekhout et al., 2011; Bordeleau et al., 2010). Higher doses were generally associated with more adverse effects, whereas low-dose regimens such as 37.5–75 mg/day appeared better tolerated (Loprinzi et al., 2000; Biglia et al., 2005). Importantly, in tamoxifen-treated patients, venlafaxine did not alter serum concentrations of tamoxifen or its active metabolites, supporting its use as a nonhormonal option with minimal CYP2D6-related interaction concerns (Sato et al., 2026). However, the included studies did not systematically evaluate blood pressure changes, serotonergic drug interactions, or rare adverse events, limiting conclusions regarding long-term safety in oncology populations.

These safety data pertain exclusively to established supportive-care and psychiatric dosing (37.5–225 mg/day). The safety and therapeutic index of venlafaxine at the substantially higher exposures that would be required for putative anticancer activity are unknown and should not be inferred from this evidence.

### 3.8 Emerging anticancer applications of venlafaxine

Two preclinical studies investigated the anticancer activity of venlafaxine, but they are not equivalent to direct antitumor evidence.

The preclinical models differ substantially in design: Niu et al. used a conventional human tumor xenograft (human melanoma cells implanted in immunodeficient nude mice) (Niu et al., 2023), whereas Zhang et al. used a syngeneic immunocompetent model (murine CT26 colon carcinoma in BALB/c mice) for *in vivo* experiments, with human colorectal cell lines (HCT116, SW480) evaluated only *in vitro* (Zhang et al., 2023). This distinction is biologically relevant given the tumor-host and neuroimmune interactions central to the norepinephrine-driven mechanism described below.

Niu et al. evaluated venlafaxine as a direct antiproliferative/pro-apoptotic agent in a melanoma model. Zhang et al., by contrast, evaluated venlafaxine specifically as an antagonist of norepinephrine-promoted colorectal cancer progression rather than as a direct cytotoxic agent (Niu et al., 2023; Zhang et al., 2023). Both studies used combined *in vitro* and *in vivo* approaches, including cancer cell lines and murine tumor models, which strengthens biological plausibility but remains insufficient for clinical inference.

Niu et al. demonstrated that venlafaxine inhibited melanoma cell proliferation, induced apoptosis, and reduced xenograft tumor growth by approximately 64% (Niu et al., 2023). These effects were associated with activation of the JNK1/2–Nur77 signaling pathway, increased reactive oxygen species generation, mitochondrial translocation of Nur77, and activation of the caspase-3/PARP apoptotic cascade.

Zhang et al. evaluated venlafaxine in a norepinephrine-driven model of colorectal cancer progression. Venlafaxine alone did not produce significant changes in tumor growth, cell viability, or angiogenic markers versus control. Its principal effect was antagonizing norepinephrine-enhanced proliferation, angiogenesis, and tumor growth: combined norepinephrine-plus-venlafaxine treatment reduced tumor volume and weight to 25.1% and 28.7%, respectively, of the norepinephrine-alone group, via inhibition of NET/SLC6A2 and the downstream NET–PPP2R1A–Akt–VEGF pathway (Zhang et al., 2023). This reflects context-dependent antagonism rather than direct venlafaxine monotherapy cytotoxicity.

The proposed molecular mechanisms identified in the preclinical studies are summarized in Figure 2.

**FIGURE 2:**
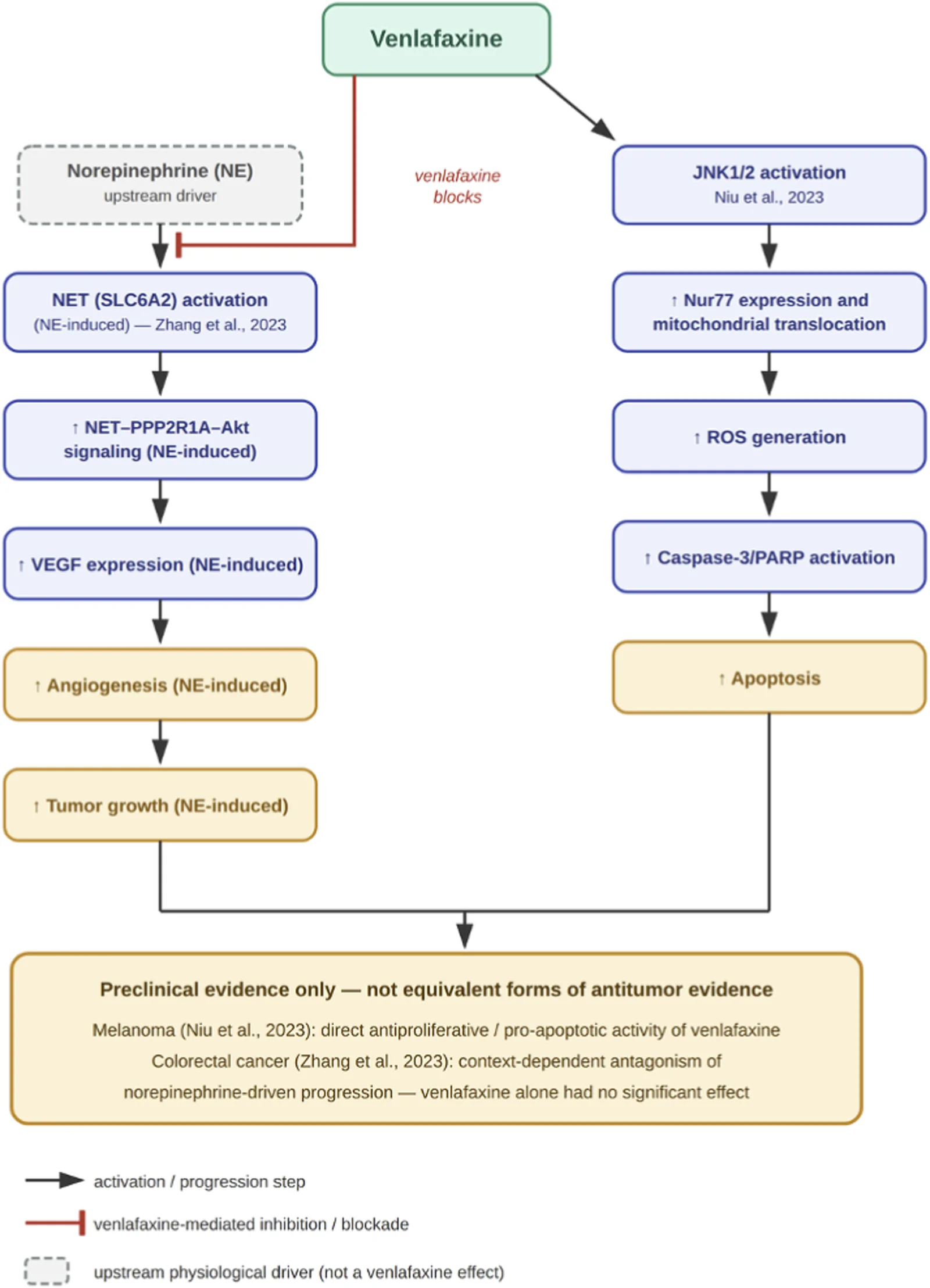
Proposed mechanisms underlying the potential anticancer activity of venlafaxine based on preclinical studies. In melanoma models, venlafaxine activated the JNK1/2–Nur77 pathway, leading to increased reactive oxygen species (ROS) generation and apoptosis. In a norepinephrine-driven colorectal cancer model, venlafaxine had no significant direct effect on tumor growth but antagonised norepinephrine-induced activation of NET/SLC6A2 and the downstream NET–PPP2R1A–Akt–VEGF pathway. These mechanisms are derived from two independent preclinical studies and have not yet been validated in clinical trials.

Collectively, these findings suggest that venlafaxine may possess previously unrecognized antitumor properties. However, the available evidence remains limited to preclinical investigations, and no clinical studies have evaluated its direct anticancer efficacy. Therefore, these findings should be considered hypothesis-generating and require further mechanistic and translational validation.

## 4 Discussion

Among the 25 included studies, the strongest clinical evidence supported venlafaxine for managing treatment-related hot flashes, whereas findings for CIPN remained inconsistent and evidence for post-surgical neuropathic pain rested on a single small trial. Evidence for other supportive care applications was limited, and two preclinical studies investigated the potential direct anticancer effects of venlafaxine with unequal evidentiary strength (see Section 3.8). Clinical and preclinical findings were synthesised and interpreted separately, and conclusions regarding anticancer activity were intentionally restricted to hypothesis-generating experimental evidence. Collectively, these findings indicate that venlafaxine has an established, evidence-based role in supportive oncology, while its potential as an anticancer agent remains experimental and requires further translational validation.

The most consistent evidence identified in this review relates to the management of vasomotor symptoms. Among the randomized, placebo-controlled trials, venlafaxine reduced hot-flash scores significantly more than placebo in one trial (Loprinzi et al., 2000; P < 0.0001 for the 75 and 150 mg doses) (Loprinzi et al., 2000), whereas in a second trial the prespecified primary endpoint at week 12 was not met for venlafaxine (P = 0.07), although a significant difference from placebo emerged over the full 12-week treatment period (P < 0.001) (Boekhout et al., 2011). Among active-comparator trials, venlafaxine was superior to clonidine for hot-flash frequency but not for severity (Loibl et al., 2007), comparable to clonidine overall (Buijs et al., 2009), comparable to gabapentin on hot-flash score while being preferred by a significant majority of patients (Bordeleau et al., 2010), and comparable to acupuncture during treatment but with less durable benefit after discontinuation (Walker et al., 2010). In the androgen-deprivation population, venlafaxine did not differ significantly from comparator at the prespecified primary endpoint (Vitolins et al., 2013). The larger reductions of approximately 50%–60% reported in the single-arm studies (Loprinzi et al., 1998; Quella et al., 1999; Biglia et al., 2005; Sato et al., 2026; van Gool et al., 2005) are within-group changes from baseline in uncontrolled designs and do not represent comparative treatment effects. These findings are clinically relevant because hot flashes represent one of the most common adverse effects of endocrine therapy and frequently contribute to impaired quality of life and reduced adherence to long-term treatment among breast cancer survivors. Effective management of vasomotor symptoms may therefore improve not only symptom control but also persistence with potentially life-saving endocrine therapy.

Importantly, the findings of the present review remain consistent with current guidance. The 2023 nonhormone therapy position statement of The North American Menopause Society continues to rate SNRIs such as venlafaxine as Level I (recommended) for vasomotor symptom management, alongside gabapentin, while explicitly advising against clonidine due to its adverse-effect profile (New Collective Author, 2023). This contemporary positioning is broadly consistent with the earlier Canadian Supportive Care in Cancer (CEPO) recommendations for breast cancer survivors specifically (L’Espérance et al., 2013), indicating that venlafaxine’s relative standing has not been superseded, though it should be considered within an evolving nonhormonal landscape rather than as a static recommendation. Notably, newer mechanistically distinct agents such as fezolinetant, a neurokinin-3 receptor antagonist, have since emerged as an additional nonhormonal option and are increasingly discussed in cancer-specific vasomotor symptom management (Zhu et al., 2025). Head-to-head comparative data between venlafaxine and fezolinetant in oncology populations are not yet available, and this represents a relevant gap for future research. The consistency between the available clinical evidence and contemporary guideline recommendations further strengthens the role of venlafaxine as an evidence-based intervention in supportive oncology.

Compared with other nonhormonal therapies, venlafaxine demonstrated efficacy comparable to gabapentin and generally similar or more rapid symptom relief than clonidine across the included studies, although adverse effects such as nausea, constipation, and appetite loss were reported more frequently in some trials. These findings are consistent with current nonhormonal treatment guidance (New Collective Author, 2023; Runowicz et al., 2016), which continues to recognize venlafaxine as one of several evidence-supported options for the management of treatment-related hot flashes in cancer survivors, alongside gabapentin and, more recently, fezolinetant (Zhu et al., 2025).

Another clinically important observation concerns the interaction between venlafaxine and endocrine therapy. Unlike potent CYP2D6 inhibitors such as paroxetine and fluoxetine, which may interfere with the metabolic activation of tamoxifen, venlafaxine appears to have minimal effects on tamoxifen metabolism. The recently published prospective study by Sato et al. demonstrated that venlafaxine significantly reduced hot flash scores without altering serum concentrations of tamoxifen or its active metabolites (Sato et al., 2026). This finding supports the preferential use of venlafaxine in patients receiving tamoxifen and is consistent with current guideline recommendations advising caution when prescribing strong CYP2D6 inhibitors to this population (Denlinger et al., 2014).

In contrast to hot flashes, the evidence supporting venlafaxine for chemotherapy-induced peripheral neuropathy remains considerably less consistent. Although several studies demonstrated clinically meaningful reductions in neuropathic symptoms, others failed to show significant benefit, particularly in the prevention of chronic oxaliplatin-induced neuropathy (Walker et al., 2010; Loprinzi et al., 2006; Taleghani et al., 2023; Irani et al., 2010). These discrepancies likely reflect substantial heterogeneity in study design, including differences in chemotherapy regimens, cumulative neurotoxic exposure, timing of intervention, treatment duration, neuropathy severity, and outcome assessment instruments. Consequently, direct comparison between studies remains challenging.

Substantial heterogeneity was also observed across studies evaluating treatment-related hot flashes. Differences in patient populations, including sex, hormonal milieu, cancer type, endocrine treatment status, venlafaxine dose, treatment duration, comparator selection, and study design, may have influenced treatment response. In particular, vasomotor symptoms experienced by women receiving endocrine therapy for breast cancer are biologically and clinically distinct from androgen deprivation–associated hot flashes in men with prostate cancer, limiting direct comparison across studies.

Comparative studies further suggest that although venlafaxine may improve neuropathic symptoms, duloxetine generally demonstrates greater and more consistent efficacy, particularly for painful CIPN. These observations are in agreement with the most recent American Society of Clinical Oncology (ASCO) Guideline Update, which recommends duloxetine as the only pharmacologic agent supported by moderate-quality evidence for the treatment of established painful chemotherapy-induced peripheral neuropathy, while concluding that evidence remains insufficient to recommend venlafaxine for either prevention or routine treatment (Loprinzi et al., 2020). Nevertheless, the favorable safety profile observed across several studies included in the present review suggests that venlafaxine may remain a reasonable alternative for selected patients in whom duloxetine is contraindicated, poorly tolerated, or unavailable. However, additional adequately powered randomized clinical trials are required before broader recommendations can be made.

The emerging evidence suggesting direct anticancer activity of venlafaxine should be interpreted within the broader context of drug repurposing. Drug repurposing has become an increasingly attractive strategy in oncology because approved medications have well-established pharmacokinetic profiles, known safety records, and substantially lower development costs and shorter development timelines than novel anticancer agents (Xia et al., 2024). Consequently, several classes of non-oncologic drugs, including antidepressants, are currently being investigated as potential anticancer therapies. Recent epidemiological evidence has further strengthened this interest. In a large propensity score-matched cohort study involving more than 420,000 participants from the UK Biobank, antidepressant use was associated with lower overall cancer morbidity and cancer-related mortality, although causality could not be established (Ma et al., 2025). While these findings should be interpreted cautiously, they support continued investigation of antidepressants as potential candidates for drug repurposing in oncology.

Within this broader context, venlafaxine has followed an unusual developmental trajectory. Originally introduced as an antidepressant, it subsequently became an established supportive care medication in oncology and is now being explored as a potential source for novel anticancer compounds.

Interest in repurposing antidepressants for oncology extends beyond venlafaxine. A recent systematic scoping review summarized extensive preclinical evidence supporting the anticancer potential of sertraline through multiple molecular pathways, while also emphasizing that translation into clinical oncology remains limited and biomarker-driven clinical trials are still lacking. These observations suggest that translational challenges are common across antidepressant repurposing research, including studies investigating venlafaxine (Blum et al., 2025).

More recently, medicinal chemistry studies have extended these observations beyond venlafaxine itself. Yuan et al. developed Nuc01, a novel venlafaxine-derived compound that combines antidepressant activity with inhibition of lysine-specific demethylase 1 (LSD1). In preclinical models, Nuc01 demonstrated antidepressant effects while simultaneously inhibiting proliferation of breast cancer cells, supporting the concept that venlafaxine-derived compounds may represent promising candidates for future anticancer drug development (Yuan et al., 2025). Importantly, Nuc01 is a structurally modified venlafaxine derivative rather than venlafaxine itself; therefore, these findings should not be interpreted as direct evidence supporting the anticancer efficacy of venlafaxine.

LSD1 has recently emerged as an important epigenetic regulator involved in tumor proliferation, metastasis, immune escape, and therapeutic resistance. Multiple LSD1 inhibitors are currently undergoing preclinical and clinical evaluation across several malignancies, highlighting the growing interest in targeting this pathway. Consequently, future development of venlafaxine-derived LSD1 inhibitors may represent an attractive translational strategy linking supportive oncology with targeted anticancer therapy (Cai et al., 2024).

Both mechanistic pathways are cancer-model-specific: JNK1/2–Nur77-dependent apoptosis has been demonstrated only in a melanoma model, and NET/PPP2R1A/Akt/VEGF-dependent antagonism of norepinephrine signaling only in a colorectal cancer model. Neither pathway has been shown to define a broadly applicable venlafaxine-sensitive cancer phenotype, and extrapolation to other tumor types is not currently supported by the available evidence.

### 4.1 Translational pharmacology: exposure-response considerations

Both preclinical anticancer studies used venlafaxine at approximately 10 μM *in vitro*, while the *in vivo* experiments employed intraperitoneal doses of 20 mg/kg in the study by Niu et al. (Niu et al., 2023) and 10 mg/kg in the study by Zhang et al. (Zhang et al., 2023). At the commonly used clinical dose of 150 mg/day, the reported steady-state Cmax of parent venlafaxine is approximately 150 ng/mL (∼0.54 μM total; ∼0.39 μM free, based on approximately 27% protein binding) (DailyMed). Clinically achieved plasma concentrations of parent venlafaxine are therefore substantially below the approximately 10 μM concentration used in the key *in vitro* anticancer experiments, with an even greater difference when free parent drug is considered.

O-desmethylvenlafaxine (ODV), the major active metabolite of venlafaxine, reaches higher circulating concentrations than the parent compound and contributes to its established SNRI pharmacological activity (DailyMed). However, the cited anticancer experiments evaluated parent venlafaxine, and it has not been established that ODV reproduces the relevant anticancer mechanisms. Accordingly, parent venlafaxine and ODV concentrations should not be summed or considered equivalent measures of anticancer exposure.

Standard body-surface-area conversion of the murine doses provides approximate human-equivalent doses of approximately 114 mg and 57 mg, respectively, for a 70-kg adult (Nair and Jacob, 2016). However, these calculations represent dose-scaling approximations and should not be interpreted as evidence of equivalent systemic exposure across species or routes of administration. In the absence of pharmacokinetic data from the experimental mouse models, the oral human dose required to reproduce the systemic or tumor exposure achieved in these studies cannot be reliably inferred.

Venlafaxine exposure within human tumor tissue has likewise not been established. These uncertainties limit direct translation of the available preclinical anticancer findings to clinically achievable dosing and underscore the need for dedicated pharmacokinetic and pharmacodynamic studies before the feasibility of anticancer dosing can be assessed.

### 4.2 Lack of clinical translation

Despite encouraging preclinical findings, translation of venlafaxine into clinical oncology has not yet occurred. Several factors may explain this gap. First, the current evidence is limited to two mechanistic preclinical studies of unequal evidentiary strength: one demonstrated direct antiproliferative and pro-apoptotic activity in melanoma models, whereas the other showed context-dependent antagonism of norepinephrine-driven tumor progression in colorectal cancer, with no significant effect of venlafaxine alone. This limited and model-specific evidence is insufficient to justify early-phase clinical evaluation. Second, the optimal anticancer dose of venlafaxine remains unknown, as the doses required for tumor inhibition may differ substantially from those used for the management of depression or cancer-related symptoms. Third, no predictive biomarkers have been identified to define patient populations most likely to benefit from venlafaxine-based therapy. Finally, no phase I or phase II clinical trials have investigated the safety, pharmacodynamics, or preliminary efficacy of venlafaxine as an anticancer agent. Consequently, the available evidence should currently be regarded as hypothesis-generating rather than practice-changing. These findings highlight an important translational gap between promising laboratory observations and clinical implementation.

### 4.3 Strengths

The present review has several strengths. To our knowledge, this is the first systematic review to comprehensively evaluate both the established supportive care applications and the emerging experimental anticancer potential of venlafaxine in oncology. Unlike previous reviews focusing exclusively on hot flashes or chemotherapy-induced peripheral neuropathy, the present review integrates evidence across multiple supportive care indications while also summarizing recent mechanistic studies investigating direct antitumor activity. Furthermore, by combining clinical and preclinical evidence, this review provides a comprehensive overview of the current knowledge and identifies important gaps requiring future investigation.

### 4.4 Limitations

Several limitations should be acknowledged. First, of the 25 included studies, most evaluated venlafaxine for the management of treatment-related hot flashes, limiting the available evidence for other oncological indications. Second, considerable clinical and methodological heterogeneity was observed across studies with respect to cancer type, patient population, intervention protocols, comparator treatments, treatment duration, and outcome assessment, precluding quantitative synthesis. Third, evidence regarding direct anticancer activity was restricted to two preclinical investigations, one of which demonstrated direct cytotoxic/antiproliferative activity, and no clinical studies have evaluated venlafaxine as an antitumor therapy. Finally, because of the limited number of available studies and their methodological heterogeneity, the findings should be interpreted cautiously and considered primarily descriptive.

A related limitation concerns population indirectness for two vasomotor-symptom trials included in this review. The Loprinzi et al. (2000) trial enrolled both women with a history of breast cancer and women without a cancer diagnosis who wished to avoid hormonal therapy because of concern about breast-cancer risk; results were not reported separately by cancer status, so the treatment effect specific to the cancer subpopulation cannot be isolated. Similarly, in the Loprinzi et al. (2006) trial (NCCTG N99C7), 61% of the 188 randomized participants had a history of breast cancer, DCIS, or LCIS, while 39% did not; the authors reported that treatment efficacy was similar between participants with and without a history of breast cancer, though disaggregated numerical results were not presented. We retained both trials because of their central role in establishing venlafaxine’s efficacy for hot flashes in the oncology population and their consistent citation as such in the broader literature; however, we flag this population indirectness as a limitation, and conclusions regarding vasomotor-symptom efficacy from these two trials should be interpreted with this caveat in mind.

Separately, this review has its own methodological limitations, distinct from limitations of the underlying evidence. The search was restricted to English-language publications, which may have excluded eligible non-English studies. We developed and circulated a methodological outline for this review among the review team on 25 June 2026, before the literature search began, and provide it as Supplementary File S1; however, this framework was not prospectively registered in PROSPERO or any other international registry, and Supplementary File S1 documents this along with the protocol amendments made during peer review. We disclose the absence of prospective registration as a limitation. Despite the re-audited and updated search strategy, we cannot exclude the possibility of incomplete retrieval, given the heterogeneous terminology used across the supportive-care and oncology literature. Finally, because narrative synthesis relies on qualitative interpretation rather than pooled effect estimates, our conclusions are inherently more subject to interpretive judgment than a quantitative synthesis would allow.

### 4.5 Future directions

Future research should focus on both clinical and translational aspects of venlafaxine in oncology. Additional adequately powered randomized controlled trials are required to further clarify its role in supportive care beyond vasomotor symptoms, particularly for CIPN and other treatment-related toxicities.

From a translational perspective, future studies should specifically prioritize: independent replication of the JNK1/2–Nur77 and NET/PPP2R1A/Akt/VEGF findings in separate laboratories; formal dose-response and exposure-response characterization to determine whether clinically achievable concentrations produce measurable antitumor activity; testing in additional tumor models beyond melanoma and colorectal cancer; evaluation using clinically relevant (oral) administration routes rather than intraperitoneal dosing; development of pharmacodynamic target-engagement biomarkers (e.g., NET occupancy, JNK1/2/Nur77 pathway activation) to confirm mechanism engagement *in vivo*; and identification of the molecular or adrenergic contexts (e.g., tumor NET expression, circulating norepinephrine levels) that may predict which tumors, if any, are venlafaxine-responsive. Only after this sequence of preclinical validation should early-phase clinical studies be considered. In addition, the rational design and optimization of venlafaxine-derived compounds, including LSD1-targeting analogs, may represent a promising strategy for future anticancer drug development.

Overall, the available evidence indicates that venlafaxine has an established, evidence-based role in the management of treatment-related hot flashes in patients with cancer, whereas its effectiveness for chemotherapy-induced peripheral neuropathy remains uncertain. Although emerging preclinical studies suggest previously unrecognized anticancer properties through multiple molecular mechanisms, these findings remain preliminary and have not yet been validated in clinical settings. Consequently, further mechanistic and translational research is required before venlafaxine can be considered a candidate for clinical evaluation as an anticancer therapy.

## 5 Conclusion

This systematic review provides a comprehensive synthesis of the available evidence regarding the role of venlafaxine in oncology, spanning established supportive care applications and emerging experimental anticancer research. The current clinical evidence most strongly supports venlafaxine as an effective nonhormonal option for the management of treatment-related hot flashes, particularly in patients receiving endocrine therapy for breast cancer. In contrast, evidence for chemotherapy-induced peripheral neuropathy remains inconsistent, with potential benefit observed in selected settings, particularly acute oxaliplatin-related neurotoxicity, but insufficient support for routine use in the prevention or treatment of chronic CIPN. Evidence for other supportive care applications, including oral mucositis-associated pain, fatigue, depressive symptoms, sleep disturbance, and quality-of-life improvement, remains limited.

Emerging preclinical studies suggest venlafaxine may exert direct antitumor effects in melanoma models via apoptosis and oxidative stress, whereas in a colorectal cancer model its effect was context-dependent antagonism of norepinephrine-driven progression rather than direct cytotoxicity. However, these findings are currently restricted to experimental models and should be regarded as hypothesis-generating rather than clinically actionable. At present, no clinical evidence supports the use of venlafaxine as an anticancer therapy.

Overall, venlafaxine has an established role in supportive oncology, particularly for vasomotor symptom control, while its potential role in anticancer drug repurposing remains an intriguing but unproven area for future research. Future studies should include adequately powered randomized trials using standardized supportive care outcomes, as well as early-phase translational investigations designed to evaluate optimal anticancer dosing, biomarker-driven patient selection, pharmacodynamic activity, and the clinical relevance of venlafaxine-derived compounds. Taken together, venlafaxine represents an established supportive care medication with promising drug-repurposing potential; however, its transition from symptom management to anticancer therapy will require substantial mechanistic, translational, and clinical validation.

## Data Availability

The original contributions presented in the study are included in the article/Supplementary Material, further inquiries can be directed to the corresponding author.
