# Supplementary material for: Venlafaxine in oncology: supportive-care evidence and emerging preclinical anticancer signals: a systematic review
Source: Front Pharmacol. 2026 Oct 16;17:1945568. doi: 10.3389/fphar.2026.1945568 (PMC13719556; doi:10.3389/fphar.2026.1945568)
Supplement: Supplementary file 1 [file Supplementaryfile1.zip › Supplementary Tables S1-3.docx]

**Supplementary Table S1. Search strategies used for the systematic literature search**

*All three databases were searched on the same date, from database inception through the search date. Full Boolean syntax, platform, filters, and yield are reported for each database to allow exact reproduction of the search.*

| **Database** | **Platform / Interface** | **Search date** | **Filters / Limits** | **Search strategy (complete Boolean syntax)** | **Records retrieved** |
| --- | --- | --- | --- | --- | --- |
| **PubMed** | PubMed (NLM) | 22 August 2026 | None | *("Venlafaxine"[Mesh] OR venlafaxine[tiab] OR Effexor[tiab]) AND (cancer[tiab] OR neoplasm[tiab] OR tumor*[tiab] OR tumour*[tiab] OR malignan*[tiab] OR carcinoma*[tiab] OR oncology[tiab] OR "breast cancer"[tiab] OR "colorectal cancer"[tiab] OR melanoma[tiab] OR "prostate cancer"[tiab] OR "head and neck cancer"[tiab])* | **219** |
| **Web of Science Core Collection** | Clarivate, Advanced Search | 22 August 2026 | None | *TS=(venlafaxine OR Effexor) AND TS=(cancer OR neoplasm OR tumor* OR tumour* OR malignan* OR carcinoma* OR oncology OR "breast cancer" OR "colorectal cancer" OR melanoma OR "prostate cancer" OR "head and neck cancer")* | **48** |
| **Scopus** | Elsevier, Advanced Search | 22 August 2026 | Language: English; Document type: Article | *TITLE-ABS-KEY(venlafaxine OR Effexor) AND TITLE-ABS-KEY(cancer OR neoplasm OR tumor* OR tumour* OR malignan* OR carcinoma* OR oncology OR "breast cancer" OR "colorectal cancer" OR melanoma OR "prostate cancer" OR "head and neck cancer") AND (LIMIT-TO(LANGUAGE,"English")) AND (LIMIT-TO(DOCTYPE,"Article"))* | **1,204** |

**Supplementary Table S2. Methodological quality assessment of included clinical studies using JBI Critical Appraisal Checklists**

*Methodological-quality categories were derived exclusively from JBI checklist item responses (randomization, allocation concealment, blinding of participants/assessors, comparability of groups at baseline, completeness of follow-up, and appropriateness of statistical analysis), independent of study outcome direction. Studies with ≥90% of applicable items rated "Yes" were categorized as High; 70–89% as Moderate–High; 50–69% as Moderate; below 50% as Low.*

| **Study** | **Study design** | **JBI tool** | **Main methodological concerns** | **Overall appraisal** |
| --- | --- | --- | --- | --- |
| Buijs et al. (2009) | Double-blind randomized crossover trial | JBI RCT | Randomization and blinding reported; crossover design appropriate; discontinuation due to adverse effects noted | High |
| Loibl et al. (2007) | Double-blind randomized trial | JBI RCT | Randomization and blinding reported; short follow-up; sample size relatively small | High |
| Loprinzi et al. (2000) | Randomized double-blind placebo-controlled trial | JBI RCT | Strong design; dose comparison included; short treatment duration | High |
| Carpenter et al. (2007) | Randomized double-blind placebo-controlled crossover trials | JBI RCT | Blinding and placebo control appropriate; modest sample size; discontinuation during follow-up | Moderate–High |
| Vitolins et al. (2013) | Randomized factorial clinical trial | JBI RCT | Randomized, double-blind, placebo-controlled, 2×2 factorial design with permuted block randomization stratified by disease severity and hot-flash severity; groups balanced at baseline; allocation concealment and blinding of treatment-delivering personnel not explicitly described; trial stopped early by the Data Safety Monitoring Board before reaching the pre-specified sample size (120 of 176 planned); adherence was significantly lower in the venlafaxine arm than in other arms (P = .009). | Moderate–High |
| Zimmerman et al. (2016) | Randomized double-blind placebo-controlled pilot trial | JBI RCT | Randomized, double-blind, placebo-controlled pilot design; groups balanced at baseline; small sample size (pilot) | Moderate |
| Bordeleau et al. (2010) | Randomized open-label crossover trial | JBI RCT | Randomized crossover design; open-label treatment may introduce performance/reporting bias | Moderate |
| Boekhout et al. (2011) | Randomized double-blind placebo-controlled trial | JBI RCT | Strong design with placebo comparator; adverse effects may have affected blinding | High |
| Biglia et al. (2005) | Prospective single-arm open-label study | JBI Quasi-experimental | No control group; open-label design; incomplete BDI assessment | Moderate |
| Irani et al. (2010) | Multicenter randomized double-blind trial | JBI RCT | Strong multicenter design; active comparators; good sample size | High |
| Durand et al. (2012) | Randomized double-blind placebo-controlled Phase III trial | JBI RCT | Strong design; small sample; limited assessable population | Moderate–High |
| Ma et al. (2023) | Phase II randomized clinical trial | JBI RCT | Randomized design; single-center; modest sample size | Moderate–High |
| Farshchian et al. (2018) | Randomized clinical trial | JBI RCT | Randomized design; limited reporting of allocation/blinding details | Moderate |
| Walker et al. (2010) | Randomized controlled trial | JBI RCT | Randomized design; different intervention types make blinding difficult | Moderate |
| Sato et al. (2026) | Prospective single-arm open-label study | JBI Quasi-experimental | No control group; small sample; short follow-up | Moderate |
| Radkhah et al. (2024) | Randomized clinical trial | JBI RCT | Randomized design; limited reporting of blinding/allocation concealment | Moderate |
| Loprinzi et al. (1998) | Prospective single-arm open-label pilot study | JBI Quasi-experimental | No concurrent control group (historical/cross-trial comparison only); no blinding; small sample (n=28) | Moderate |
| Quella et al. (1999) | Prospective single-arm open-label pilot study | JBI Quasi-experimental | No concurrent control group (historical/cross-trial comparison only); no blinding; small sample (n=16–21) | Moderate |
| Loprinzi et al. (2006, N99C7) | Randomized, open-label, three-arm phase III trial (two-arm analysis reported) | JBI RCT | Central randomization with concealed allocation; groups balanced at baseline; open-label design (oral capsules vs. IM injection blinding not feasible); analysis restricted to patients with complete booklets rather than full ITT; enrolled a mixed cancer/non-cancer population (see Limitations, Section 4); third arm discontinued early due to slow accrual, explicitly accounted for | Moderate |
| Kus et al. (2016) | Retrospective case–control study | JBI Case-Control | Treatment (venlafaxine) was not randomly assigned but selected based on clinician-identified depression, introducing confounding by indication; case/control groups not matched; chemotherapy-regimen composition differs substantially between arms (Table 1) without statistical adjustment; retrospective design | Moderate |
| Taleghani et al. (2023) | Double-blind, placebo-controlled, three-arm trial (as reported) | JBI RCT | Double-blind, placebo-controlled, three-arm design; small sample size (n=41); short treatment duration (4 weeks) with 2-month follow-up; randomization method and allocation concealment not described in the abstract; details regarding who was blinded were not reported in the abstract. | Moderate |
| Tasmuth et al. (2002) | Randomized double-blind placebo-controlled crossover trial | JBI RCT | Randomized, double-blind design using inactive placebo with identical tablets supplied by the manufacturer, who also performed the randomization; method of sequence generation and allocation concealment not described; crossover design appropriate for chronic stable pain, but no washout period between treatment periods is described and carryover effects were not assessed; blinding of treatment-delivering personnel not explicitly reported; analysis restricted to the 13 patients completing the protocol rather than all 15 entered, with no intention-to-treat analysis, and the highest-dose comparison based on 11 patients; very small sample with no reported sample-size calculation | Moderate |
| van Gool et al. (2005) | Prospective single-arm open-label clinical case series | JBI Quasi-experimental | No control group and no blinding; no predefined referral or inclusion criteria (the authors state that no strict referral criteria were established); no predetermined criteria for treatment response, with the decision to continue or stop taken jointly by patient and clinician; hot flush frequency self-monitored over short 2–3 day periods without a validated instrument, and flush severity not assessed; concomitant clonidine continued in one patient; no statistical analysis performed, with results reported descriptively; only 14 of 22 referred women started treatment | Low |

### **Supplementary Table S3. Risk of bias assessment of preclinical animal studies using the SYRCLE Risk of Bias tool**

*The SYRCLE tool appraises only the in-vivo/animal component of each preclinical study. Where a study also reports in-vitro mechanistic experiments (e.g., cell-line assays), the SYRCLE judgment should not be interpreted as an overall quality rating for the study as a whole; in-vitro components were not formally appraised due to the absence of a validated standard tool for this purpose.*

| **Domain** | **Niu et al., 2023** | **Zhang et al., 2023** |
| --- | --- | --- |
| Sequence generation | Unclear | Low |
| Baseline characteristics | Low | Low |
| Allocation concealment | Unclear | Unclear |
| Random housing | Unclear | Unclear |
| Blinding of caregivers/investigators | Unclear | Unclear |
| Random outcome assessment | Unclear | Unclear |
| Blinding of outcome assessment | Unclear | Unclear |
| Incomplete outcome data | Low | Low |
| Selective outcome reporting | Low | Low |
| Other sources of bias | Low | Low |
| **Overall risk of bias** | **Moderate** | **Moderate** |
