# Supplementary material for: Venlafaxine in oncology: supportive-care evidence and emerging preclinical anticancer signals: a systematic review
Source: Front Pharmacol. 2026 Oct 16;17:1945568. doi: 10.3389/fphar.2026.1945568 (PMC13719556; doi:10.3389/fphar.2026.1945568)
Supplement: Supplementary file 1 [file Supplementaryfile1.zip › SYSTEMATIC REVIEW PROTOCOL.docx]

**SYSTEMATIC REVIEW PROTOCOL**

**Venlafaxine: a potentially emerging anticancer drug**

**Revised manuscript title:**
Venlafaxine in Oncology: Supportive-Care Evidence and Emerging Preclinical Anticancer Signals: A Systematic Review

**Protocol date:** 25 June 2026
**Reporting framework:** PRISMA 2020

**1. Background and rationale**

Drug repurposing has emerged as an increasingly important strategy in oncology because it allows the investigation of established medications with known pharmacological and safety profiles for potential applications beyond their originally approved indications. Antidepressants have attracted particular interest in this context because accumulating experimental evidence suggests that some agents may influence biological pathways involved in cancer development and progression.Venlafaxine is a serotonin–norepinephrine reuptake inhibitor (SNRI) primarily approved for the treatment of depressive and anxiety disorders. In oncology, venlafaxine has also been investigated for the management of cancer- and treatment-related symptoms, particularly vasomotor symptoms associated with endocrine therapy. Additional clinical studies have explored its effects on chemotherapy-induced peripheral neuropathy (CIPN), treatment-related pain, and other supportive-care outcomes. Alongside these established or emerging supportive-care applications, experimental studies have begun to investigate whether venlafaxine may exert direct anticancer effects. Proposed mechanisms include modulation of apoptosis, oxidative stress, tumor-cell proliferation, angiogenesis, and intracellular signaling pathways. The extent and translational relevance of this evidence remain uncertain. The initial methodological outline for this systematic review was developed on 25 June 2026 with a primary focus on the potential anticancer and drug-repurposing properties of venlafaxine. During development of the review, the scope was broadened to incorporate clinically established and emerging supportive-care applications of venlafaxine in oncology, allowing clinical supportive-care evidence and experimental anticancer evidence to be evaluated within a single systematic framework while remaining analytically distinct. The review was therefore designed to systematically identify, critically evaluate, and narratively synthesize the available evidence regarding the role of venlafaxine across the oncology continuum, distinguishing clinical supportive-care applications from experimental evidence of direct anticancer activity.

**2. Review objectives**

The primary objective of this systematic review is to systematically evaluate the available clinical and preclinical evidence regarding the use and potential biological effects of venlafaxine in oncology.

The specific objectives are:

1. To evaluate clinical evidence regarding the effectiveness and safety of venlafaxine for the management of cancer- and cancer treatment-related symptoms, including vasomotor symptoms, chemotherapy-induced peripheral neuropathy, pain, and other supportive-care outcomes.
2. To identify and characterize experimental evidence investigating potential direct anticancer effects of venlafaxine.
3. To summarize the molecular and biological mechanisms proposed to underlie the reported anticancer effects of venlafaxine.
4. To critically evaluate the methodological quality and risk of bias of the available clinical and preclinical evidence.
5. To identify gaps between experimental findings and clinical translation and define priorities for future research.

**3. Protocol and registration**

The methodological framework for this systematic review was initially developed on **25 June 2026**. The review was conducted and reported in accordance with the principles of the **Preferred Reporting Items for Systematic Reviews and Meta-Analyses (PRISMA) 2020 statement**.

The protocol was **not prospectively registered in PROSPERO or another public systematic-review registry**. The methodological framework is therefore provided as a supplementary review protocol to improve transparency and reproducibility. Any modifications made to the initial review plan during development of the systematic review or following peer-review reassessment are described in the **Protocol Amendments** section below.

**4. Eligibility criteria**

Studies were considered eligible if they investigated venlafaxine in patients with malignant diseases or in experimental cancer models and evaluated either supportive-care outcomes or potential direct anticancer effects.

**4.1 Population and experimental models**

Eligible clinical studies included adult patients with a diagnosis of malignant disease, including patients undergoing active cancer treatment and cancer survivors experiencing cancer- or treatment-related symptoms.

**For vasomotor symptom (hot-flash) outcomes, an eligibility amendment permitted studies enrolling mixed populations of women with a history of breast cancer and women without a cancer diagnosis who were unwilling or unable to use hormonal therapy because of breast-cancer-risk concerns. This amendment reflected the design of two influential hot-flash trials included in the final review (Loprinzi et al., 2000; Loprinzi et al., 2006). Where available, cancer-specific results were considered separately; otherwise, the mixed population was recognized as a source of indirectness.**

Eligible preclinical studies included experimental cancer models investigating the effects of venlafaxine on tumor-related biological or molecular outcomes.

**4.2 Intervention**

The intervention of interest was **venlafaxine administered or investigated as an independently assessable intervention**.

Studies evaluating venlafaxine in combination with other interventions were eligible only when the independent contribution of venlafaxine could be determined.

**4.3 Outcomes**

Two distinct categories of outcomes were considered.

**Supportive-care outcomes** included:

- vasomotor symptoms, including hot flashes or hot flushes;
- chemotherapy-induced peripheral neuropathy and treatment-related neurotoxicity;
- cancer- or cancer treatment-related pain;
- oral mucositis-associated pain;
- fatigue;
- depressive symptoms;
- sleep-related outcomes;
- quality of life;
- other clinically relevant cancer treatment-related symptoms or toxicities.

**Direct anticancer outcomes** included:

- tumor-cell proliferation or viability;
- apoptosis;
- tumor growth or progression;
- migration and invasion;
- angiogenesis;
- oxidative stress;
- tumor-associated molecular signaling pathways;
- metastatic behavior;
- other experimentally measured antitumor effects.

Clinical supportive-care outcomes and experimental anticancer outcomes were planned to be synthesized and interpreted separately to avoid extrapolating preclinical antitumor findings to clinical cancer treatment.

**5. Study designs**

Eligible clinical evidence included:

- randomized controlled trials;
- randomized crossover trials;
- non-randomized comparative studies;
- prospective interventional studies;
- retrospective observational studies;
- other original clinical studies providing independently assessable data on venlafaxine in an oncology population.

Eligible preclinical evidence included experimental studies evaluating venlafaxine in cancer models and reporting direct tumor-related biological outcomes.

Reviews, systematic reviews, meta-analyses, editorials, letters, commentaries, protocols, and publications without original outcome data were excluded from the primary evidence synthesis.

Conference abstracts without sufficient methodological and outcome information for reliable assessment were excluded.

Studies conducted exclusively in non-cancer populations were excluded.

Studies in which the independent effect of venlafaxine could not be determined were excluded.

**6. Information sources**

The following electronic databases were searched:

- PubMed/MEDLINE;
- Scopus;
- Web of Science Core Collection.

The databases were searched from inception to the prespecified search date and subsequently updated during revision to identify newly published and potentially missed eligible studies.

**7. Search strategy**

The search strategy was designed to identify studies evaluating venlafaxine in oncology, including both supportive-care applications and potential direct anticancer effects. The search combined the generic and trade names of venlafaxine (venlafaxine, Effexor) with terms relating to cancer and oncology, including neoplasm, tumor/tumour, malignancy, carcinoma, oncology, and named cancer types, as appropriate for each database. Separate supportive-care, symptom, or cancer-treatment terminology was not used as a search concept; studies addressing supportive-care indications were captured through the cancer and oncology terms and were subsequently assessed against the eligibility criteria at the title/abstract and full-text stages.

During revision, the search strategy was re-audited and the searches were re-executed in all three databases to ensure that the reported strategy corresponded exactly to the search as performed. The complete database-specific search strategies exactly as executed, including database/platform, search date, search fields, Boolean operators, and any limits or filters applied, are reported in Supplementary Table S1. The final updated search was conducted on 22 August 2026.

**8. Study selection**

All records retrieved from the electronic databases were combined and duplicate records were removed before screening. Retracted publications identified during the search process were excluded from the evidence base and documented in the PRISMA study-selection process.

Study selection was conducted in two stages.

First, titles and abstracts of retrieved records were screened against the eligibility criteria. Records clearly unrelated to venlafaxine use in oncology or the predefined clinical or experimental outcomes were excluded.

Second, the full texts of potentially eligible reports were retrieved and assessed against the complete eligibility criteria.

Reasons for exclusion at the full-text stage were documented. A study-specific list of full-text reports excluded from the systematic review, together with the corresponding reason for exclusion, is provided in the Supplementary Material.

The complete study-selection process is reported using a **PRISMA 2020 flow diagram**

**9. Data extraction**

Data were extracted using a structured data-extraction framework.

For clinical studies, the following information was collected where available:

- first author;
- year of publication;
- country;
- study design;
- cancer type;
- participant characteristics;
- sample size;
- cancer treatment context;
- venlafaxine dose and treatment duration;
- comparator or control intervention;
- supportive-care indication;
- outcome definitions and assessment instruments;
- main efficacy findings;
- adverse events;
- relevant methodological characteristics.

For preclinical studies, the following information was extracted where available:

- first author;
- year of publication;
- cancer type;
- experimental model;
- cell line or animal model;
- venlafaxine concentration or dose;
- duration of exposure;
- comparator/control;
- tumor-related outcomes;
- effects on proliferation, apoptosis, migration, invasion, tumor growth, angiogenesis, or other relevant endpoints;
- proposed molecular mechanisms;
- principal findings.

Where information was not reported in the published article, it was recorded as not reported/not available rather than inferred.

**10. Methodological quality and risk-of-bias assessment**

The methodological quality of included clinical studies was assessed using the appropriate **Joanna Briggs Institute (JBI) Critical Appraisal Checklists** according to study design.

The JBI assessment considered relevant domains such as randomization, allocation procedures, baseline comparability, blinding, completeness of follow-up, outcome measurement, statistical analysis, and other design-specific methodological considerations.

Preclinical animal evidence was assessed using the **SYRCLE Risk of Bias tool**, including domains relating to sequence generation, baseline characteristics, allocation concealment, random housing, blinding, random outcome assessment, incomplete outcome data, selective outcome reporting, and other potential sources of bias.

Methodological assessments informed interpretation of the strength and reliability of the evidence rather than automatically excluding studies solely on the basis of an overall quality category.

**11. Data synthesis**

Because substantial clinical and methodological heterogeneity was anticipated across cancer populations, supportive-care indications, venlafaxine doses, comparator interventions, study designs, experimental models, and outcome definitions, the primary synthesis was planned as a **structured narrative synthesis**.

Clinical supportive-care evidence was synthesized separately according to the principal clinical indication, including:

- vasomotor symptoms;
- chemotherapy-induced peripheral neuropathy/neurotoxicity;
- cancer treatment-related pain and mucositis;
- other supportive-care outcomes.

Experimental studies investigating potential direct anticancer activity were synthesized separately according to cancer model, experimental setting, reported antitumor effects, and proposed molecular mechanisms.

A quantitative meta-analysis was to be considered only if a sufficiently homogeneous group of studies was identified with respect to population, intervention, comparator, and outcome assessment. Where substantial heterogeneity precluded meaningful quantitative pooling, findings were synthesized narratively.

Clinical supportive-care findings and experimental anticancer findings were not quantitatively combined.

**12. Assessment of heterogeneity**

Clinical heterogeneity was evaluated qualitatively by considering differences in:

- cancer type and disease setting;
- patient characteristics;
- cancer treatment exposure;
- indication for venlafaxine;
- venlafaxine dose and duration;
- comparator;
- follow-up duration;
- outcome definitions and measurement instruments.

Methodological heterogeneity was evaluated according to study design and methodological quality.

For preclinical evidence, heterogeneity was considered in relation to cancer model, experimental conditions, venlafaxine exposure, outcome measures, and proposed biological mechanisms.

**13. Reporting of results**

The systematic review was planned to report:

1. the PRISMA 2020 study-selection flow diagram;
2. characteristics of included clinical and preclinical studies;
3. clinical findings organized according to supportive-care indication;
4. experimental evidence of direct anticancer activity;
5. proposed molecular mechanisms;
6. methodological quality/risk-of-bias assessments;
7. limitations of the available evidence;
8. the current translational gap between experimental anticancer findings and clinical application.

Particular caution was planned in the interpretation of experimental antitumor evidence. Preclinical findings were to be described as hypothesis-generating and were not to be interpreted as evidence supporting clinical use of venlafaxine as an anticancer treatment in the absence of corresponding clinical evidence.

**14. Protocol amendments**

The initial methodological outline was developed on **25 June 2026** and focused primarily on evaluating the potential anticancer and drug-repurposing properties of venlafaxine.

During development of the systematic review, the scope was broadened to incorporate clinical evidence regarding the established and emerging supportive-care applications of venlafaxine in oncology. This amendment provided a more comprehensive assessment of venlafaxine's role across the oncology continuum. We subsequently maintained clinical supportive-care evidence and experimental anticancer evidence as distinct evidence streams throughout the synthesis and interpretation.

Following peer-review feedback, the manuscript title was revised to “Venlafaxine in Oncology: Supportive-Care Evidence and Emerging Preclinical Anticancer Signals: A Systematic Review.” This revised title was considered more accurate to the composition and strength of the available evidence and to avoid implying that venlafaxine has established clinical anticancer activity.

The literature search was re-audited and updated in August 2026, and the database searches were rerun across PubMed/MEDLINE, Scopus, and Web of Science Core Collection using the database-specific strategies reported in Supplementary Table S1.

We reassessed studies identified through the updated search using the same substantive eligibility framework, and updated the study-selection process, PRISMA flow diagram, evidence tables, methodological quality assessments, and narrative synthesis as appropriate.

During eligibility reassessment, we clarified the clinical population criterion for vasomotor-symptom studies to permit the two specified mixed cancer/non-cancer hot-flash trials (Loprinzi et al., 2000; Loprinzi et al., 2006). This amendment reflects the design of these influential trials and is explicitly reported in the final manuscript.

The updated review also expanded reporting of the PRISMA audit trail by providing a study-specific list of reports excluded after full-text assessment, together with reasons for exclusion.

These amendments are reported to provide transparency regarding differences between the initial methodological outline and the final systematic review.

**15. Registration and protocol availability**

The systematic review was **not prospectively registered in PROSPERO**.

The initial methodological outline was developed on **25 June 2026**, and the present supplementary protocol documents the review framework and subsequent amendments made during conduct and revision of the review. The absence of prospective registration is acknowledged to ensure transparent reporting of the review methodology.
